# Implications of graphene-based materials in dentistry: present and future

**DOI:** 10.3389/fchem.2023.1308948

**Published:** 2024-02-29

**Authors:** M. Roma, Shreya Hegde

**Affiliations:** ^1^ Manipal College of Dental Sciences, Mangalore, Mangalore, Karnataka, India; ^2^ Manipal Academy of Higher Education, Manipal, Karnataka, India

**Keywords:** graphene, graphene oxide, dentistry, dental implants, osseointegration, bone regeneration, dental prosthesis, health

## Abstract

Since the advent of nanoscience, nanobiomaterials have been applied in the dental industry. Graphene and its derivatives have attracted the most interest of all of them due to their exceptional look, biocompatibility, multiplication differential, and antibacterial capabilities. We outlined the most recent developments about their applications to dentistry in our review. There is discussion of the synthesis processes, architectures, and characteristics of materials based on graphene. The implications of graphene and its counterparts are then meticulously gathered and described. Finally, in an effort to inspire more excellent research, this paper explores the obstacles and potential of graphene-based nanomaterials for dental aspects.

## 1 Introduction

Dental health is crucial as the oral conditions have a big impact on people’s health and quality of life (Lamster, 2021). But according to the World Health Organisation (WHO), more than 70% of the population globally suffered mouth ailments in 2016 (Gordon and Donoff, 2016; Lamster, 2021). The 74th World Health Assembly of the WHO, held in 2021, focused heavily on oral wellbeing (Lamster, 2021). The most frequent dental fricative diseases are dental caries, periodontal issues, missing teeth, and malignancies of the mouth (Li et al., 2022). Nowadays, maintaining one’s teeth clean might be challenging. There is still no perfect treatment for oral problems, despite the fact that many different techniques and strategies have been employed. These methods have been improved by the use of an extensive range of biomaterials.

Tissue degeneration brought on by trauma, infections, or tumours is one of the most frequent conditions in the dental field, notably bone degeneration (Liu et al., 2020). Numerous initiatives are now aimed at repairing tissue problems. Dental tissues take longer time to recuperate as cemental regeneration is slow and pulp regeneration is difficult. Alveolar bone healing is also reasonably active and fast (Liu et al., 2020). The development of tissue engineering, which is widely viewed as a superior therapeutic strategy, required the use of scaffolds. The bulk of commercially available biomaterials lack the osteoinductive properties necessary for bone regeneration today (Wu et al., 2017). So it is mandatory to find an osteoinductive biomaterial for osseous healing.

Due to their many benefits, dental implants are frequently used to replace missing teeth in the area of dentistry. It is widely acknowledged that osseointegration represents the pinnacle of dental implant success. Dental implant materials have historically been made of titanium and its alloy because to their high biocompatibility and mechanical properties. The adoption of Ti and its alloy as implant substructures has been attributed to their high biocompatibility, mechanical properties, and other qualities. Despite all of its advantages, titanium implants might fail because of poor osseointegration. As a result, it’s critical to enhance the functionality of Ti dental implants, and changes to the implant surface have a significant impact in this regard (Steflik et al., 1999). Many different bioengineered substances are used to ameliorate the osteogenic qualities of dental implants. Additionally, the primary cause of dental implants failing is peri-implantitis (Hirooka and Renvert, 2009). Therefore, it is crucial to research novel, good antibacterial dental implant surfaces.

Nanomaterials have demonstrated outstanding abilities to increase the durability and wear resistance of tooth fillings and sealants. Moreover, the application of restorative materials using nanoparticles demonstrated good antibacterial characteristics (Sharan et al., 2017). Outstanding nanoparticles are used frequently in the dentistry disciplines of restorative materials, adhesives, cements, primers, and more because of the aforementioned benefits.

**FIGURE 1:**
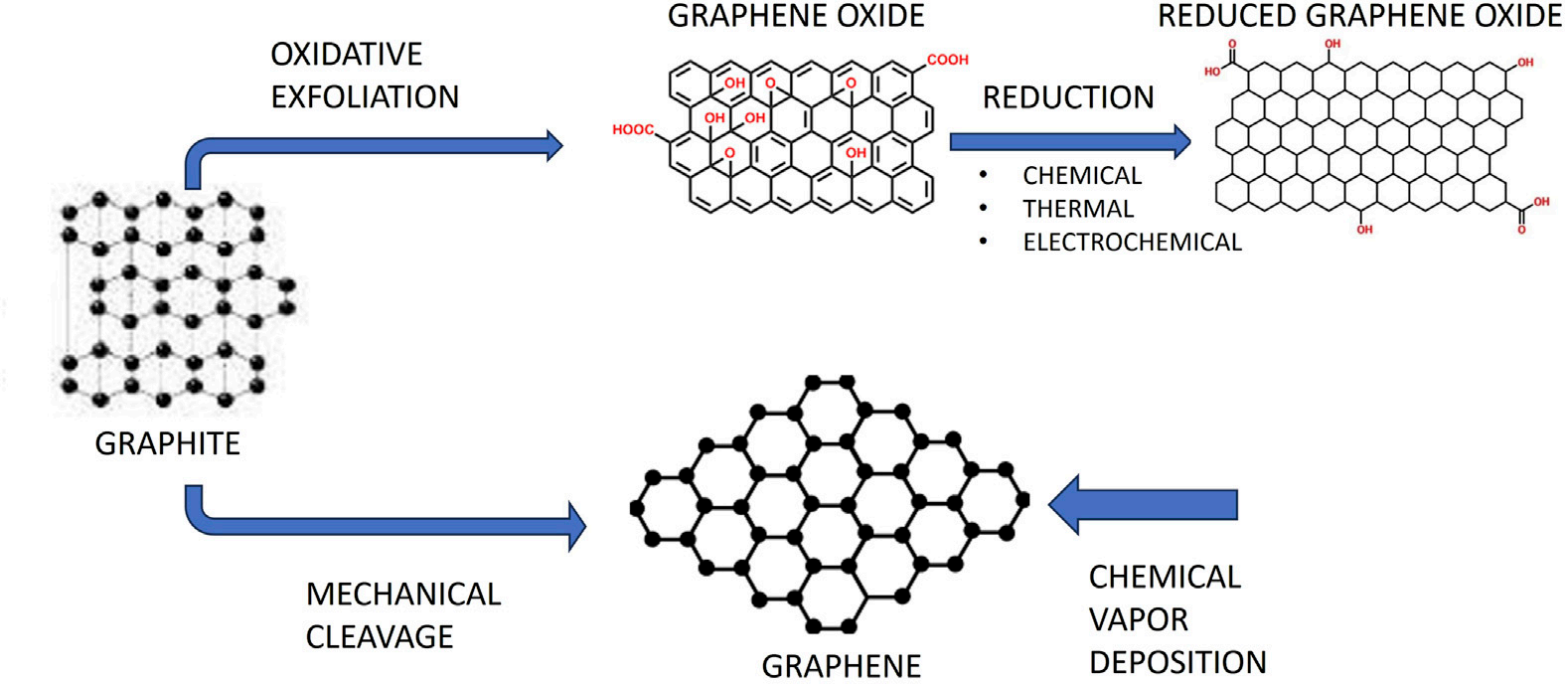
The structures of graphene-based materials.

The strongest and thinnest of the many nanomaterials is graphene, a promising 2D carbon-based nanomaterial. Using mechanical exfoliation and adhesive tape, Novoselov and Geim isolated it for the first time in 2004. In 2010, they were honoured with the Nobel Prize (Novoselov et al., 2004). Four categories of graphene-based materials might be distinguished: graphene oxide (GO), reduced graphene oxide (rGO), single-layer graphene, and few-layered graphene (Figure 1) (Bei et al., 2019). Due to their outstanding biocompatibility, excellent electrical conductivity, and flawless physical qualities, graphene and its derivatives have gained a lot of interest in the medical and biomedical disciplines. A lot of focus has also been placed on graphene and its derivatives in the disciplines of dentistry and other fields.

Noteworthy development has been instituted over the years in terms of controlling the characteristics of graphene and its co-derivatives, illuminating their fundamental operations, and expanding the range of possible applications. Although there have been many great reviews released, the majority of them have generally concentrated on one particular feature. This paper bestowes an architecture of the numerous types, properties, and uses of graphene-based materials in order to demonstrate current advancements. The goal of this research was to provide an overview of the dental applications of materials based on graphene as well as obstacles and future opportunities. Graphene nanoparticles can adapt to the complicated oral milieu, which includes high masticatory force and oral bacteria colonisation, thanks to their numerous unique mechanical and physiochemical features. Research on graphene nanoparticles in dentistry is moving quickly, particularly in the areas of implant coatings, periodontitis treatment, and caries. Graphene with its outstanding properties like high optical translucency, high thermal conductivity and mechanical strength, high flexible thin film, high electronic mobility, and high surface area makes it superior to other nanomaterials used in dentistry. Graphene-based materials have shown significant promise in the last 20 years in the fields of nanobiotechnology and nanomedicine. These applications include biosensors, photothermal and photodynamic treatment options, drug delivery, tissue engineering, implants, and antibacterial materials. The majority of these applications are directly tied to dentistry. Several studies have shown that graphene’s surface may be chemically modified with polymer compounds, nanoparticles, and small molecules. This property makes graphene more appropriate for applications such as photothermal therapy for cancer treatment, drug transport, and imaging of cells and tumours. Research has demonstrated that few-layer graphene (FLG), which generally consists of one to six layers, may boost the biomechanical and physicochemical characteristics of biomaterials while also being noncytotoxic and biocompatible.

## 2 Graphene and its derivative synthesis and structure

The strongest and thinnest substance at the moment is graphene, a potential 2D carbon-based nanomaterial that is of single atom thickness. NbO_2_ (GO) and CxHyOz (rGO) are the two most popular graphene co-derivatives. Although graphene and its by-products have indistinguishable arrangement, they have various functional groups, which might account for the variations in their physical and chemical characteristics.

### 2.1 Graphene synthesis and structure

The Novoselov and Geim group originally isolated graphene in 2004 using a sticky tape and mechanical exfoliation Novoselov et al. (2004). A honeycomb-like lattice of carbon atoms that have undergone sp2 hybridization makes up graphene. Six-membered rings piled parallel make up its structure, and there are no chemical groups on its surface Zhang et al. (2019). Good mechanical stability, extensive surface area, excellent conductivity, and other properties, graphene attracted a lot of interest (Du et al., 2020).

Graphene produced by mechanical exfoliation has a very low yield while being highly pure and defect-free. Numerous synthesis techniques have been created in an effort to increase graphene yield. The top-down technique and the bottom-up approach are the two primary synthesis methods (Huang et al., 2012; Liao et al., 2018). On the one hand, the bottom-up strategy involves producing graphene directly from carbon materials using techniques including chemical vapour deposition (CVD), graphitization of carbon incorporated substratum through torrid heat annealing, and solid-phase deposition (Guo et al., 2009; Xiao et al., 2011). Contrarily, the top-down approach makes use of micromechanical cleavage, liquid-phase exfoliation, and chemically-assisted GO exfoliation before reduction treatment.

#### 2.1.1 Mechanical exfoliation

In 2004 (Novoselov et al., 2004), the Geim group made the first self assembled graphene via mechanical exfoliation. It is possible to mechanically exfoliate graphene using a sticky tape consisting of graphite crystals. Then, after the tape is maneuvered with particular solvents (such as acetone), graphene was extracted and desorbed (Phaedon and Christos, 2012). Although there were no chemical groups or flaws in the finished graphene, the yield was relatively low (Phaedon and Christos, 2012).

#### 2.1.2 Liquid-phase exfoliation

This is an effectual way to make graphene on a small scale. A suspension of graphite is first created in an organic solvent in order to reduce the van der Waals tensions between the graphite layers. Then, using ultrasonic at a specific voltage, the graphite was separated into sheets of graphene. Substantial amounts of mono- and multilayer graphene were created following centrifugation (Ghuge et al., 2017). Graphene is tiny and pure, but the number of layers is unpredictable. In addition, the use of organic solvents and surfactants pollutes the environment. The process of exfoliating graphene makes it challenging to get rid of the remaining surfactants. The three organic solvents that are most frequently utilised are dichlorobenzene (DCB), N-methyl-2-pyrrolidone (NMP), and N-dimethyl-formamide (DMF). However, they are harmful to cells and poisonous (Liao et al., 2018).

#### 2.1.3 Chemical vapor deposition

In order to produce finest monolayer or few-layered graphene with minimal price and good qulaity, one of the most efficient technologies, CVD, has been widely used. On the metal, a sizable monolayer graphene coating developed (Ming et al., 2018). The manufacture task involves warming up methane, ethane, or propane to an intense heat, followed by pyrolyzing it to produce C onto metal foils consisting of Cu, Ni, Fe, Pt, and Ru. The graphene layer then worked from the unbound C atoms (Liao et al., 2018).

#### 2.1.4 Chemical exfoliation

Chemical approaches are one of the most effective ways to create materials based on graphene among the many techniques. The initial technique for creating GO is called the Hummers process, which demands for ultrasonic treatment of graphite with H₂SO₄, NaNO_3_, and KMnO_4_ in water. Then, using reducing agents, GO is converted to rGO. Finally, heat or chemical processes transform rGO into graphene. However, it is challenging to eliminate all the molecules in the GO that include oxygen. Additionally, the lengthy processing durations and hazardous gases like NO_2_ and N_2_ O_4_ are detriments to the synthesis process.

#### 2.1.5 Epitaxial graphene

On the SiC wafers, epitaxial graphene may be produced under conditions of extreme vacuum and high heat. Throughout the procedure, Silica atoms are sublimated on SiC wafer surface and C atoms are preserved on the SiC wafer surface, eventually creating C6H6 (Norimatsu and Kusunoki, 2014). However, in contrast to conventional exfoliation techniques, the as-prepared graphene is not homogeneous due to the simultaneous development of graphene in several positions.

Additionally, Nickel diffusion is a viable substitute for SiC crystals. A graphene-like lattice structure was achieved for nickel by evaporating a Ni coating onto a SiC crystal. As the surface is sintered to high temperature, the carbon disseminates into and out of the nickel layer to form a layer of graphene. These techniques make it simpler to separate the graphene layer from the SiC crystal (Park and Ruoff, 2009).

It is most likely that three-dimensional fracture deflection, bridging, and sheet pull-out mechanisms are responsible for the improved mechanical properties of bioceramics brought about by the deliberate addition of graphene family elements. Because of its two-dimensional sheet-like structure, graphene and ceramic grains can have a larger contact area and, potentially, a stronger connection, which slows the spread of cracks at grain boundaries. It has been shown that graphene, GO, and rGO can all be successfully dispersed in ceramics despite having distinct chemical properties. Crucially, when it comes to the creation of ceramic composites, graphene can tolerate extreme processing conditions such high temperatures (up to 1,150°C) and pressures. It should be mentioned that GO is reduced to rGO *in situ* by high temperatures.

Composites with improved mechanical properties can be created by combining materials and polymers linked to graphene. To obtain good dispersion within various polymers, one can choose different graphene derivatives; for instance, water-soluble polymers can be easily treated using graphene oxide (GO). Similar to ceramic composites, higher interfacial adhesion between phases is likely facilitated by the enormous surface area of graphene’s 2D sheet-like structure, which also makes fracture toughness and crack deflection easier. Interestingly, the improvements are still noticeable in the polymer matrix at modest filler loading. The addition of 2 weight percent graphene oxide (GO) nanosheets to polyvinylidene difluoride (PVDF) resulted in a 92% increase in tensile strength and a 192% rise in Young’s modulus (Park and Ruoff, 2009).

In addition to the improvements in mechanical and physical characteristics, the combination of polymers with graphene can enhance bioactivity and encourage stem cell development. For example, compared to the unmodified mat, the addition of GO to electrospun polylactic-co-glycolic acid (PLGA) nanofibrous mats improved the adsorption of the osteogenic inducer dexamethasone. In the presence of dexamethasone, this composite enhanced the expression of the collagen I, ALP, and OCN genes in MSCs. Composites containing rGO and poly-dopamine (PDA) have the ability to cause hydroxyapatite to nucleate when submerged in simulated bodily fluid. In comparison to glass, the rGO/PDA-based surfaces also encouraged increased osteoblastic cell attachment and proliferation.

## 3 Dentistry-related properties and its derivatives

### 3.1 Biocompatibility and cytotoxicity

Evaluation of cytotoxicity and biocompatibility of these materials is crucial (Olteanu D. et al., 2015). The biocompatibility concerns of these materials are of prime concern among the researchers. Concentrations, surface functionalization, and other parameters were the affected ones up until this point.

The biological suitability and cytotoxicity of graphene and related derivatives are dose-dependent, according to numerous studies. Some studies demonstrated that GO < 20 g/mL, the effect of GO on fibroblasts was minimal (Khan et al., 2019). GO, however, became more hazardous to cells when the concentration reached 50 g/mL. When Wang et al. (2011) studied the cytotoxicity of GO in mice, they found there was harmful effects. There was no evidence of harm at GO concentrations of 0.1 and 0.2 mg. Mice exhibited chronic toxicity at a dosage rise to 0.4 mg.

Studies have also examined how surface functionalization affects cytotoxicity. By Diana et al., it was established that GO, N-Gr, and TRGO were hazardous to dental follicle stem cells (Olteanu D. et al., 2015). According to the findings, TRGO had the highest cytotoxicity while GO had the lowest. Malgorzata et al. contrasted the vitality of leukocytes when treated with GO, rGO, and rGO-PEG (Podolska et al., 2020). Leukocyte viability at a concentration of 50 g/mL was not significantly different, according to the results, proving that surface functionalization had no impact on cellular viability.

The protein interactions would cause inflammation when the biomaterials were transplanted into the tissue. Many variables, including surface charge, topography, and chemical compositions, played a role in this process and had an impact on the protein resorption. In addition, a variety of chemicals, including betaines, were crucial in the development of inflammation (D’Onofrio et al., 2019). Additionally, the tissue inflammation brought on by products made of graphene should be given a lot of consideration. According to Eriberto et al., the soft and bony tissue surrounding dental implants may become chronically inflamed as a result of titanium nanoparticles produced from the implants (Bressan et al., 2019).

Therefore, we should pay attention to the impact on the inflammation of the surrounding tissue when employing graphene nanomaterials as coatings for dental implants and other devices. Rosa et al. (2021) study shown that friction occurs when dental implants are placed under weights greater than 400 mN. They also looked into whether or not macrophages’ increased expression of inflammatory markers like TNF- was caused by graphene nanocoatings. Of course, more research is needed to support this finding.

### 3.2 Cellular differentiation stimulation

Biomaterials that are best suited for regenerative medicine have the aptness to promote cell adhesion, multiplication, and differentiation. Literature search have elicited that graphene and its co-derivatives have the capability to undergo a variety of differentiations, including osteogenic transformation and dental pulp regeneration.

Studies have shown that the osteogenic maturation of many kinds of cells, such as MC3T3-E1, BMSCs, PDLCs, DPSCs, etc., can be triggered by graphene-based materials Lee et al. (2015a), Xie et al. (2017). Graphene, GO, and rGO have all been put to the test for osteogenic differentiation using various synthesis techniques and form factors. To stimulate osteogenic differentiation, DPSCs, PDLCs, DFPCs, and BMSCs are employed Han et al. assessed the potential of DPSCs to trigger osteogenic differentiation after employing the CVD process for creating a monolayer of graphene on copper foils (Xie et al., 2017). After 14 and 28 days of incubation, the outcome demonstrated that the osteogenic proteins and RUNX2, OCN, and COL were increased on graphene.

To investigate whether these materials may repair tooth pulp, some research has enthralled on the neuronic proliferation of G-based nanomaterials. Seonwoo et al. (2018) created NFs using an electrospinning approach and added rGO and polycaprolactone (PCL) before examining how this improved DPSC neurogenesis. The findings demonstrated that Tuj-1, an early marker of neurogenesis, and NeuN, a late marker of neurogenesis, were highly expressed in NFs with rGO. Kohei et al. (2018) used the GO reformed COL sponge scaffold to study the regenerative process of periodontal tissue and revealed the formation of new bone.

### 3.3 Antibacterial property

Low cytostatic and multiplication differential are essential for a great biomaterial in dentistry. Antibacterial properties cannot be disregarded aside from these. Hu et al. (2010) made the initial discovery of the antibacterial property of graphene-based compounds. According to Gholibegloo et al. (2018), GO, GO-Car, and GO-Car/HAp can each reduce the multiplication of S. mutans by 67%, 86.4%, and 78.2%, respectively. To examine its antibacterial properties, numerous composites had been created. Some researchers also created dental adhesive, glass ionomer cements, and PMMA using graphene-based materials (Bregnocchi et al., 2017; Sun et al., 2018).

Furthermore, to increase its antibacterial action, graphene has been functionalized with a variety of nanomaterials, including polymers, enzymes, metal ion/oxide NPs, and photocatalytic materials. Graphene has recently been employed as a vehicle for the regulated release of traditional antibiotics, resulting in increased effectiveness for therapy and reduced toxicities. Moreover, a synergistic impact between graphene and other nanomaterials has led to the development of several multicomponent materials that have enhanced antibacterial activity. The creation of new graphene-based materials, their interactions with biomolecules, their cytotoxicity, their *in vivo* toxicity, and their uses in antibacterial activity, drug transport, wound healing, and coating materials have all seen significant advancements in recent years (Sun et al., 2018).

## 4 Graphene-based materials and its applications in dentistry

**FIGURE 2:**
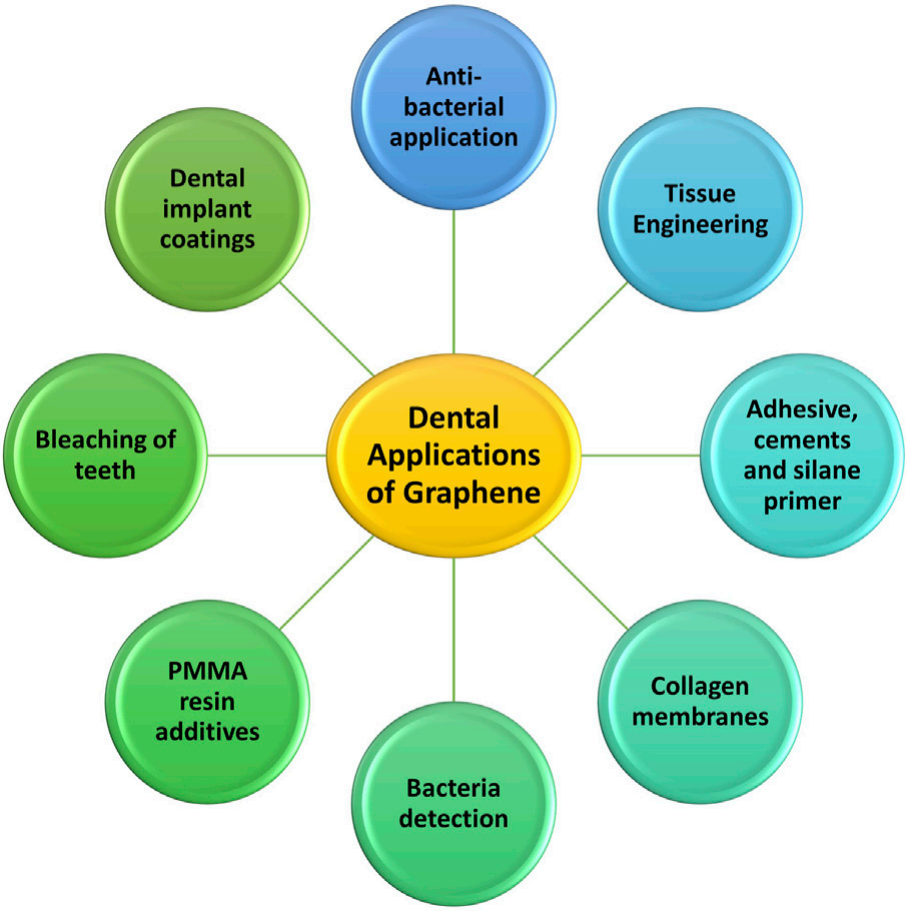
Dental applications of graphene and its derivatives.

The following list and discussion of numerous uses is based on the increased varieties of graphene-based materials, upgraded synthesis techniques, and tailored features (Figure 2).

### 4.1 Regenerative medicine/tissue engineering

Numerous literature have shown that graphene-based materials can help diverse cells, like MC3T3-E1, PDLCs and DPSCs, to differentiate into osteoblasts. Regarding osseous engineering, numerous graphene-based products (such as composite substances, scaffolds, and surface coatings) may be employed (Rosa et al., 2016). Numerous studies have suggested that graphene may encourage various stem cell types to develop into osteoblasts. According to Fan et al. (2014), the G/HAp composite sheet showed excellent Biomimicking mineralization. Xie et al. (2017) have demonstrated that osteogenic differentiation is similar. They used CVD to create monolayer graphene, after which they assessed the level of mineralization and the expression of proteins and genes related to ossification. They discovered that graphene increased the expression of OPN and OCN in DPSCs and encouraged the expression of RUNX2 and OCN. Additionally, Li et al. (2009) demonstrated that Graphene might increase the expression of genes associated to osteogenesis (OCN, OPN, BMP-2, and Runx2) when compared to a control. Additionally, the high protein-level expression of OCN has further supported the improved osteogenic effect.

rGO coating was successfully created by Kim et al. (2017) on biphasic calcium phosphate (BCP). The findings demonstrated that as compared to the control, the rGO groups had a higher rate of new bone volume regeneration (Wu et al., 2019). Different functionalizations of GO, including scaffolds and nanosheets, had been done to affirm the osteogenic differentiation of GO. Phosphorene is a modern 2D nanomaterial that has gained significant attention since graphene. When compared to materials made of graphene, phosphorene demonstrated excellent biodegradability and biocompatibility. Its features were relatively close to those of C6H6 -based materials. Liu et al. (2019) also looked into how GO and phosphorene worked together to promote osteogenic differentiation.

There were additional observations of the neuronal and odontogenic proliferation brought on by C-12 based materials. The neural development of SCAP can be influenced by graphene dispersion (Jelena et al., 2018). The graphene dispersion group discovered nice cell bodies via a protracted process. Seven days following nerve induction, the graphene dispersion group displayed significant levels of NF-M and III-tubulin expression as well as strong immunoreactivity to NeuN and III-tubulin, demonstrating that graphene facilitated SCPAs’ neural development. In order to further the neuronal maturation of C-12 based materials, Seonwoo et al. (2018) produced an NFs mixed with rGO and PCL by electrospinning process and explored the increased neuroplasticity of DPSCs (Olteanu E. D. et al., 2015). According to the findings, Tuj-1 and NeuN were highly expressed in NFs with 0.1% and 1% rGO, but NeuN was only intensely expressed in NFs with greater rGO concentrations. Rosa et al. (2016) looked at how GO affected the differentiation of DPSCs in order to demonstrate the neuronal differentiation of GO. With the odontogenic multiplication of DPSCs, GO also greatly increased DMP-1 and DSPP in addition to the high expression of Runx2 and OCN.

As is well known, periodontitis is an inflammatory condition that causes severe damage to periodontal tissues such the cementum, alveolar bone, and periodontal ligament. As periodontitis worsened, the tooth faced the possibility of being lost, which caused numerous functional issues. As a result, regeneration is very important and has attracted numerous researchers. GO demonstrated a hydrophilic surface and good dispersibility in comparison to graphene and rGO, which enabled the absorption of several related proteins. In a 3D COL sponge scaffold with GO dispersion experiment, Kawamoto et al., the histometric study revealed that the amount of newly produced bone in the GO group was 2.7 and 2.3 times more than in the control group, respectively (Dreanca et al., 2020). Neo osseous structure was discovered and filled the furcation defect in an *in vivo* investigation. Even more intriguingly, cementum-like tissue that resembled a periodontal ligament was also found in the GO group. The GO and silk-fibroin composites were created by Vera-Sánchez et al. (2016), who also assessed how well they promoted osteogenic development and cementoblast differentiation.

### 4.2 Adhesives, cements and silane primer

Two types of often used materials in dental restorations are adhesives and cements. Even though they demonstrated the benefits of aesthetic appeal and high hardness, their development was constrained by issues with excessive polymerization shrinkage and poor antibacterial properties. The silane primer was crucial to the bonding of the zirconia.

Due to its many benefits, graphene and its by-products have ameliorated the characteristics of adhesive adhesives (Farooq et al., 2021). Due to their antibacterial and antibiofilm properties, graphene nanoplatelets (GNPs) are frequently manufactured as fillers for dental adhesives made of polymer. It has been demonstrated that the nanocomposites containing GNPs may efficiently suppress S. mutans cells without reducing the bonding efficiency (Bregnocchi et al., 2017). GNPs may therefore be the perfect filler for dental adhesives because their antibiofilm activity did not affect their mechanical capabilities.

When two distinct types of calcium silicate cements were mixed in varying amounts with graphene nanosheets in powder form, the resultant GNP-cement composites performed well in terms of reducing the bonding time and raising the hardness of both cements. However, one cement called Endocem Zr (ECZ) had dramatically worsened bonding capabilities, showing that while the addition of GNPs may upgraded the physiomechanical characteristics of materials (Nizami et al., 2020).

Fluorinated graphene (FG), which is dazzling white, may be a superior filler in dentistry compared to grey GNPs. When utilised to modify GICs, FG offers significant benefits in terms of their mechanical, tribological, and antibacterial properties. In comparison to conventional GICs, composites not only improve compressive strength and Vickers micro hardness, but also reduce friction coefficient. When it comes to antibacterial capabilities, the GIC/FG composites successfully combat *Staphylococcus aureus* and *Streptococcus* mutans (Sun et al., 2018).

The mechanical qualities of composite—ZrO_2_ bonded resin, silane primers’ exhibited poor adhesive layer (Fallahzadeh et al., 2017). To enhance the mechanical qualities of the adhesive layer, incorporation of GO sheets with silane primers is advised. The findings demonstrated that the addition of GO sheets enhanced surface roughness, marginally increased the water contact angle, and greatly boosted the shear bond strength of composite—ZrO_2_ bonded resin (Khan et al., 2019). In light of this, materials based on graphene provide perfect fillers for adhesives, cements, and silane primers.

### 4.3 Polymethyl methacrylate resin (PMMA)

Over the years, PMMA has been utilised in prosthodontics, particularly in fabrication of Cds and RPDs. It has several benefits, including ease of manufacture, economical, low MOE, ease of repair, and good aesthetics (Bacali et al., 2020). However, PMMA still has drawbacks, including poor biofilm prevention, weak mechanical qualities, and significant polymerization shrinkage (Ruse and Sadoun, 2014; Matsuo et al., 2015). The graphene family has recently demonstrated favourable antibacterial and good mechanical properties in various forms in other sectors. Azevedo et al. (2019) have rehabilitated the maxillary arch by introducing graphene oxide (GO) into the PMMA resin because of the high mechanical strength. There were no mechanical, cosmetic, or other issues discovered 8 months later, proving that adding GO to PMMA resin would be a wise choice for prosthodontic reformation. PMMA containing graphene-silver nanoparticles (Gr-Ag) was reported by Bacali et al. (2020) The composites’ mechanical characteristics, hydrophilic qualities, and shape were further assessed and the findings were remarkably greater than those of the pure PMMA group. Additionally, Bacali and his colleagues evaluated the bactericidal capabilities of Gr- Ag-modified PMMA and the outcomes demonstrated that all Gram-negative strains, including S. aureus, *Escherichia coli*, and S. mutans, showed better inhibitory effect in Gr-Ag-modified groups (Bacali et al., 2020). In conclusion, materials based on graphene might be the best filler to enhance PMMA’s physical-mechanical and antibacterial capabilities.

Meanwhile, Lee and his colleagues have utilised nGO to enhance the antimicrobial-adhesive properties of PMMA resin (Lee et al., 2018a). After cultivating C. albicans for 28 days, PMMA with 2% nGO had stronger anti-adhesion effects demonstrating the hydrophilicity of PMMA may be increased by the addition of nGO. By incorporating G-AgNp, Bacali et al. (2020) assessed the overall properties of PMMA resin like biocompatibility, mechanical properties, etc. The findings indicated that PMMA had a worse antibacterial impact on *S. aureus* than the G-AgNp-containing group, which showed good antibacterial effects on both Gram-positive and Gram-negative strains. As a result, the graphene family has shown promise as a filler when combined with PMMA for antibacterial applications.

### 4.4 Dental implant coatings

Due to their numerous benefits, including their superior mechanical properties, corrosion resistance, and good biocompatibility, Ti and its alloys have been extensively applied in dental implants (Xie et al., 2014; Jeong et al., 2017). Implant non success still occurs due to ineffective osseointegration and tendency to produce peri-implantitis (Berglundh et al., 2019; Kordbacheh et al., 2019). As a result, numerous surface alterations using materials based on C-12 are exploited to enhance the bioactivities of titanium and its alloys (Barfeie et al., 2015; Chouirfa et al., 2019).

It is commonly recognised that osseointegration is the benchmark for dental implants’ success. Consequently, development of new bone between bone tissues and dental implants is very important. The modification of dental implants received a lot of interest due to graphene’s improved osteogenic differentiation in bone tissue creation. According to Park et al. (2017) review, there are basically four different types of graphene-based modification strategies: Layer-by-layer assembly, PMMA-mediated approach, electrophoretic deposition, and APTES-induced method are among the techniques used.

Numerous researchers have worked hard to promote the osteogenic qualities. Gu et al. (2014) used a PMMA-based technique to successfully create mono-coat G sheets on Ti substrates. The outcome shown that graphene sheets outperformed the control in terms of adhesion and proliferation of hGFs, hASCs, and hBMMSCs.

After pretreatment with APTES, Jung and his colleagues created a Dex-loaded rGO coating on Ti13Nb13Zr (MPCR-TNZ) multipass rolled Ti alloy, demonstrating stable long-term release behaviours of Dex (Jung et al., 2015). One such mechanism is π-π stacking. In conclusion, the Dex-loaded rGO-MPCR-TNZ improved MC3T3-E1 cell proliferation and facilitated osteoblast differentiation. In conclusion, materials based on graphene are a strong contender for dental implant surface modification materials that, when used properly, can enhance the osseointegration of implants.

Additionally, dental implant surface coatings have generated a great deal of interest due to graphene’s exceptional antibacterial properties. It is widely acknowledged that bacterial infections continue to play a role in dental implants failing. Therefore, it is imperative to modify the titanium surface to make it antimicrobial. Qian et al. (2018) studied the effect of electrostatically created GO coating loaded with minocycline onto Ti and found that *S. aureus*, *Streptococcus* mutans, and *E. coli* could not thrive on GO-modified surfaces.

### 4.5 Bleaching of teeth

As is well known, hydrogen peroxide (H_2_O_2_) has long been used extensively for in-office whitening. The bleaching procedure can be carried out by the H_2_O_2_ molecules penetrating deeply into the teeth. However, the relatively high quantities of H_2_O_2_ had certain undesirable effects, such irritated gums and sensitive teeth (Carey and Clifton, 2014). When compared to H_2_O_2_ alone, Su et al. (2016) found that a (Co)/TPP)/rGO nanocomposite was more effective at whitening teeth stained by dyes and tannins. In addition, the active free radical produced by H_2_O_2_ has a very little lifetime. Therefore, H_2_O_2_ must first enter the teeth and swiftly form active free radicals in order to have a strong bleaching impact. To speed up the bleaching process, additional counteractions between the staining molecules and H_2_O_2_ can be generated using the Co/TPP/rGO nanocomposite as a catalyst. In conclusion, materials based on graphene show promise as a catalyst for tooth whitening applications when used in the right types and concentrations.

### 4.6 Antibacterial property

Dental caries, periodontitis, and peri-implantitis all arise as a result of the creation of bacterial biofilms (Berglundh et al., 2019). Numerous novel techniques for preventing the production of biofilms were investigated. Hu et al. (2010) made the initial discovery of the antibacterial property of graphene-based compounds.

Periodontitis and peri-implantitis can both be treated with photodynamic therapy (PDT), a different approach. In conjunction with PDT, Pourhajibagher et al. (2019) looked at the impact of graphene quantum dot (GQD)-curcumin (Cur) on perio-pathogen biofilms. Reactive oxygen species (ROS) were produced by GOD- Cur-PDT with a dose-dependent propensity. Additionally, the expression of the rcpA genes from A. actinomycetemcomitans, fimA genes from P. gingivalis, and inpA genes from P. intermedia was decreased by 8.1, 9.6, and 11.8 fold, respectively.

Additionally, several researchers created GICs, PMMA, and dental adhesives using graphene-based materials to enhance their physical characteristics and antibacterial capacity (Bregnocchi et al., 2017). It’s interesting to note that Sun et al. (2018) tested the antibacterial effect of GIC/FG composites on *S. aureus* and S. mutans, finding that FG (4 wt%) had the maximum antibacterial activity for both organisms at 88.1% and 85.3%, respectively.

### 4.7 Fungal growth inhibition

A frequent factor in dental implant failure is peri-implantitis. Additionally, 31% of the peri-implantitis locations had *Candida* albicans, which rapidly gained widespread attention (Tatullo et al., 2019a). Patients with peri-implantitis had five times more species of *Candida* albicans than healthy people (Alrabiah et al., 2019). Additionally, antifungal medications frequently fail due to *Candida* albicans’ high level of resistance. A useful way to stop the growth of biofilms is to modify dental implant coatings. Agarwalla et al. (2021) created a graphene nanocoating to test the ability of *Candida* albicans biofilms to inhibit growth twice (TiGD) and five times (TiGV). The XTT reduction experiment revealed that the absorbance of the TiGD and TiGV groups was lower than that of the control group. Following that, the colony-forming unit assay revealed less viable yeast units in the TiGD and TiGV groups at all time periods, demonstrating that graphene has an inhibitory effect on the production of fungal biofilms.

### 4.8 Biosensor for biomarker detection from saliva

The detection of dental illnesses can enhance patient quality of life and lower mortality rates for some critical conditions. Due to their excellent electrical and mechanical properties, graphene-based materials are frequently utilised to diagnose tooth diseases (Goldoni et al., 2021).

#### 4.8.1 Identification of bacterial and viral markers

On tooth enamel, Mannoor et al. (2012) created the first graphene nano-sensors in 2012. They created a wireless readout coil linked to a silk fibroin and a graphene sensing element that was then applied to tooth enamel. By self-assembling AMP-graphene peptides onto the graphene, the precise biological recognition was obtained. The binding of a single *E. coli* on the naked graphene nanosensor was visible from the decrease in electrical resistance. The detection and wireless remote monitoring of *Helicobacter pylori* in saliva were made possible by the AMP-modified graphene nanosensor that demonstrated a good correlation between peptides and bacteria. The electrochemical platform was created by Gandouzi et al. utilising rGO and gold nanoparticles, and the sensor shown remarkable sensitivity to the markers (Ioannidis et al., 2019). Lee et al. (2015a) created sandwich-type biosensors to identify the human odontogenic ameloblast-associated protein (ODAM) in order to diagnose periodontal disease in its early stages (Wu et al., 2018). In order to detect the human papillomavirus type 16 (HPV-16), Chekin et al. (2018) created a rGO/MoS 2 glassy carbon electrode, demonstrating its great stability and storage performance.

#### 4.8.2 Detection of drugs

The body fluid saliva can be used to check for drugs and other dangerous chemicals. Using a biosensor to identify the analytes of drugs and dangerous substances is an excellent idea. Materials based on graphene are being used to create the portable biosensors. For instance, Mohamed and his collaborators developed a bio-sensing platform to identify the medications antipyrine and benzocaine. They coated the GO sheets with metal nanoparticles to boost the biosensor’s selectivity, resulting in high repeatability and good selectivity (Mohamed et al., 2017). Parate and other researchers designed electrochemical biosensors with graphene to detect the byproducts of smoke and tobacco with a broad linear range of 1–100 nM and the sensitivity of 1.89 A/decade (Parate et al., 2019).

#### 4.8.3 Cancer biomarker detection

For patients, diagnosis at the preliminary stage is very crucial. A biological substance known as a biomarker can detect the presence of diseases like infections and malignancies (Henry and Hayes, 2012). Interleukin-8 (IL-8) overexpression has been linked to the advancement of tumours in cases of oral cancer. To detect IL-8 in saliva, Verma and his colleagues created a biosensor using ITO glass that had been treated with rGO and coated with Au NPs (Verma et al., 2017). The biosensor manifested good reproducibility with long-term stability. The biosensor’s retention rate after 3 months of dry storage is 94.3%. Even after 4 months of dry storage, the biosensor’s performance was maintained at 91.8%. Graphene-based equipment is regarded as the pinnacle of technology in the biosensor sectors due to its exceptional electrical characteristics. Today, graphene and borophene share a comparable anisotropic characteristic (Tatullo et al., 2019b). The effectiveness of biosensors will therefore be significantly increased when graphene is combined with appropriate 2D nanomaterials. Prior to their final clinical application, this may also be a positive trend in the development of graphene-based biosensors.

### 4.9 Neutralization of enamel and dentin demineralization

Common side effects with orthodontic treatment is a white spot lesion (WSL), which is brought on by enamel surface demineralization (Bishara and Ostby, 2008). Therefore, overcoming WSL during orthodontic treatment is quite important. To stop enamel demineralization brought on by bacteria, several researchers are now concentrating on the discovery of novel bonding agent composites. Because of GO’s strong antibacterial properties, Lee and his colleagues incorporated it to a bioactive glass (BAG) (Lee et al., 2015a). The duration of the GO group’s anti-demineralization lengthened as GO concentrations rose. Additionally, after 24 and 48 h, GO-containing groups also shown greater antibacterial action. The synergistic effect of GO’s antibacterial action and BAG’s ion-releasing function may be responsible for the composites’ anti-demineralization mechanism. To sum up, GO is a promising addition for properly combating enamel demineralization.

Dentin demineralization brought on by acids from bacteria, food, and surroundings was linked to dental caries and dental erosion, resulting in painful and sensitive dentin (Addy, 2005). Five distinct functionalized GO nanocomposites were created by Nizami et al. (2020), and their biological and demineralization preventive properties were assessed. The dentin slices coated with GO-Ag, GO-Ag-CaF2, and GO-CaF 2 all displayed superior decalcification prevention when compared to the control. Additionally, when compared to other groups, the GO-Ag and GO-Ag-CaF2 groups demonstrated greater antibacterial activity, which may be explained by the synergistic impact of GO and Ag. Additionally, the f-GO coatings on the dentin surface exhibit minimal colour fluctuation, demonstrating the potential of GO as a dentin anti-demineralization resistant material.

### 4.10 Collagen membranes

COL membrane is frequently utilised as a barrier membrane in guided bone regeneration (GBR) and guided tissue regeneration (GTR) to prevent the invasion of soft tissue by new bone (Elgali et al., 2017). Even though the COL membrane has many beneficial characteristics, such as ease of manipulation and minimal surgical intervention, it still requires a number of alterations to increase biocompatibility (Chu et al., 2017b). Marco et al. (2017) were able to enrich the collagen membranes with GO through the interconnection of oxygenated carbon functional elements with COL through H_2_ bonding (Moldovan et al., 2023). Reduced deformation capacity, more roughness, and increased stiffness were all characteristics of the GO-enriched membranes. The cell proliferations of hGFs were substantially higher than the control after 3 days of incubation on membranes containing 2 and 10 g/mL GO. In terms of the inflammatory response, at day 3, cells cultivated on GO-coated membranes demonstrated considerably reduced release of IL-6 and PGE2 than the control. Radunovic et al. affirmed the supercilious cell proliferations on the GO-coated membranes at days 14 and 28 with respect to DPSCs (Rai et al., 2020).

### 4.11 Drug delivery

Dental caries, endodontic, and periodontal illnesses all interact with bacteria in a close way. There are typically a number of bacterial groups present, necessitating a coordinated antibacterial approach. In Asia and Europe, amoxicillin (AMOX), is the antibiotic commonly used for the treatment of periapical infections. The dose in the conditional paste is not well controlled (Nan, 2016). Medication carriers can readily reach effective medication concentrations in the infected site by realising the progressive release of antibiotic medicines. According to Trusek and Kijak (2021), GO had the capacity to operate as a drug carrier, particularly in the treatment of oral ailments. Leu-Leu-Gly, a peptide linker, was used to join the AMOX and GO, and it was then disseminated in the hydrogel. Enzymatic hydrolysis of AMOX demonstrated its efficient release and the suppression of bacterial strain development.

## 5 Challenges and perspective

**FIGURE 3:**
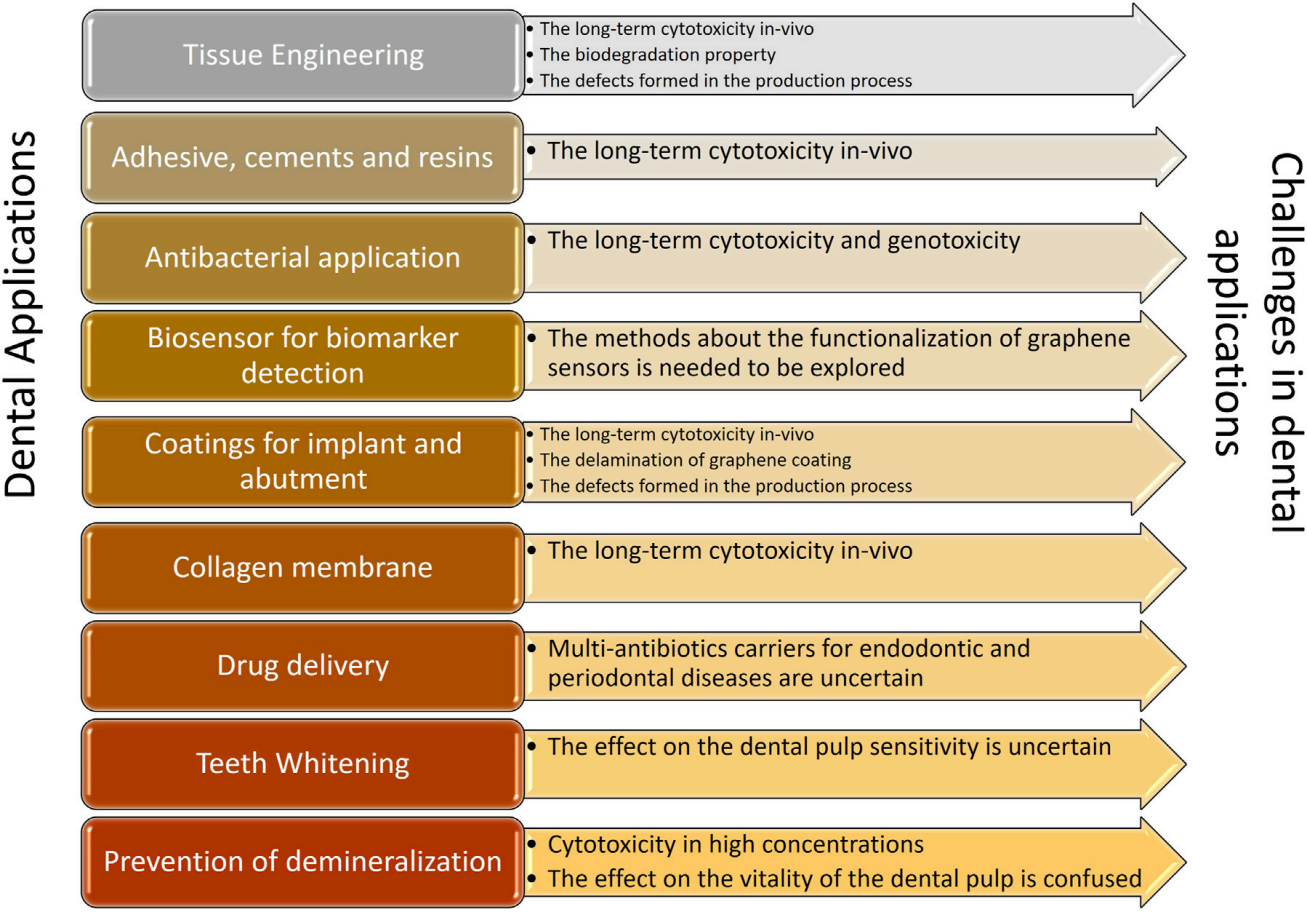
Applications and challenges of graphene in dentistry.

Due of its ability to differentiate cells and act as an antimicrobial, graphene and derivatives are employed in dental research. This study summarised current developments in broadening the categories of graphene-based materials and the research on features relevant to dentistry, enhancing our understanding of these categories. When compared to other research in the field, it demonstrated a more thorough and complete overview of significant advancements in dental applications, including osseous regeneration, dental implant coatings, antibacterial characteristics, and COL membranes. In addition to aforementioned applications, a lot of emphasis has been laid on some brand-new areas including drug delivery, enhancing remineralization strategies, developing biosensors for the detection of oral biomarkers, and stopping the growth of fungi. The biosensor could be utilised to detect bacterial and viral markers, medication markers, and cancer indicators with the usage of graphene nanoparticles. Prior to the complete commercialization of applications of graphene in dentistry, there are still several obstacles to be overcome (Figure 3).

## 6 The approach to address degradation

Biodegradation presents an additional challenge for products made from graphene, particularly in the field of tissue engineering. The ideal biomaterial should not include any harmful compounds when new tissue forms. There is currently little published research on the biodegradation of products based on graphene. To address this issue, graphene-based material should be investigated as the optimal biomaterial.

### 6.1 The long-term cytotoxicity *in vivo*

The top-notch biomaterials ought to be both *in vitro* and *in vivo* biocompatible and free of long-term cytotoxicity. Uncertainty regarding its cytotoxicity *in vitro* and *in vivo* as well as its probable causes is a significant obstacle in clinical applications due to our poor understanding of graphene and its derivatives. According to numerous research, there is currently no consensus regarding the cytotoxicity and potential risks of materials based on graphene. Concentrations, surface functionalization, varieties of the graphene family and synthesis techniques, and the number of layers are among the variables that affect cytotoxicity. There is currently no consensus regarding the upper limit concentration despite the fact that numerous facts have concentrated on the dose-dependent influence on cytotoxicity (Duch et al., 2011). The processes underlying cytotoxicity may be greatly influenced by ROS. Regarding the synthetic processes, graphene sheets made using the CVD approach were shown to be biocompatible without overt cytotoxicity. The cytotoxicity of cells may, however, increase when graphene is disseminated in solution. This could be due to buildup or sharp-edge penetration into the cells. As a result, we anticipate seeing an increase in both *in vitro* and *in vivo* investigations for long-term biocompatibility.

### 6.2 Strategy to resolve the biodegradation

Biodegradation is common with graphene-based materials, particularly in regenerative medicine. The ideal biomaterial should not contain any substances that could be hazardous during the development of new tissue. There is currently a dearth of literature on the biodegradation of materials based on graphene. To address this issue, graphene-based materials should be investigated and given consideration as excellent biomaterials.

### 6.3 Flaws elicited during the production process

Although the graphene utilised in the current investigation was a controlled, defect-free sample that was free of contamination, the synthesis quality should nevertheless be closely scrutinised when it was employed in a clinical setting. Factually, variations in synthesis techniques are primarily to blame for the range of unanticipated faults that have arisen. The qualities, such as susceptibility and electrical structure, will alter if the flaws develop (Nan, 2016). Therefore, studying how to prevent flaws from forming during the creation of graphene would be a difficult yet fruitful research subject.

### 6.4 Negative regulation of cell cycle

Few recent research have specifically examined how GO affects the cell cycle. Currently, Hashemi et al. (2020) were creative in concentrating on the impact of GO on the cell cycle. DNA synthesis is a critical step in cell division. The increase in DNA synthesis during the S phase of the cell cycle may be brought on by specific mutagenic substances. According to some underlying mechanisms in Hashemi et al. (2020) study, including DNA damage, ROS formation, and double-strand breaks in the DNA, GO boosted DNA synthesis. In mGO and nGO, cell apoptosis was higher and showed concentration- and size-dependent effects. The G2/M phase in the GO groups has been blocked, according to the cell cycle results. Therefore, prior to the final therapeutic application, the impact of GO on the cell cycle should be carefully studied and investigated.

### 6.5 Delaminated graphene coatings

Although graphene has several benefits for the dentistry industry, its clinical application still requires careful thought. Materials based on graphene have mostly been used as coatings for dental implants and surfaces for tissue engineering. Friction could lead to the delamination of carbon-based coatings on titanium when utilised as dental implant coatings with stresses greater than 400 mN (Rosa et al., 2021). According to Rosa et al. (2021), there was no discernible difference between SRP and the control after stimulating the SRP and pig maxilla to test the integrity of graphene nanocoatings. However, the coverage area in ROI C from the bone group was reduced by 35%. Consequently, considerable consideration should be given to graphene delamination (Rosa et al., 2021). It is important to carefully research how to increase the bonding strength of graphene-based materials and derivatives. Currently, layer-by-layer self-assembly procedures and spin coating techniques are the primary physical methods used to apply graphene-based materials coatings to titanium surfaces. The chemical reactions involving graphene and titanium still face technological challenges. The physical combination is less effective and more unstable than chemical techniques.

Consequently, the delamination of graphene coatings when utilised as coatings is a risk factor. According to some experts, the soft tissue and bone around dental implants may experience persistent inflammation as a result of titanium nanoparticles produced from the implants. Therefore, emphasis should be placed on the delamination of graphene coatings on the inflammation of the surrounding tissue when using graphene-based nanomaterials as coatings for dental implants.

### 6.6 Upgraded strategies about the functionalization of graphene sensors

The vast surface area of graphene-based nanomaterials offered good adhesive conformability when utilised as biosensors, and the functionalization of graphene for biological recognition can be accomplished through AMP-graphene peptide. But there are several types of germs in the mouth. As a result, further ways need be developed to identify more bacteria, and functionalizing graphene as a biosensor is crucial.

### 6.7 Sole antibiotic transporter for endo- perio diseases

AMOX, a broad-spectrum antibiotic that may be used to treat tooth ailments, had been successfully delivered via GO as a drug carrier. The constraint, however, is that additional research is required before it can be determined whether multiple medications might be transported in unison. Eventually, the dental industry will be very interested in graphene and its derivatives for a very long period. Graphene, as a more dependable and environmentally acceptable biomaterial, has the potential to lead to more efficacious dental therapies hencefoward, despite the fact that there are some limits in the practical clinical use of dentistry.

### 6.8 The unfavourable antibacterial effect on the polymicrobial strains

Since extensive research has been conducted on the antibacterial effects of graphene on single bacterial strain or monoclonal biofilm, but lacks the knowledge on mature polymicrobial biofilms. Based on the aforementioned restrictions, there is still a long way to go before graphene-based materials in the dental professions are finally used in clinical settings.

*In vivo* research, where quantities and alterations of materials studied can significantly alter the observed effects, likewise exhibit a lack of agreement. Mice given intravenous injections of GO at a dose of 20 mg/kg can produce micronucleated polychromic erythrocytes (Hashemi et al., 2020), while at a dose of 25 mg/kg, there might not be any adverse effects on reproduction. Therefore, rather than making generalised observations, it is crucial to discriminate between the many material variants and experimental settings when drawing conclusions about the character of graphene toxicity and biocompatibility. The safety characteristics of particular graphene-based and modified materials under circumstances pertinent to their planned clinical usage will also be the main focus of future study. Apart from the biological issues, there are additional areas that need further clarification, like the materials’ long-term stability. This is particularly problematic for GO-based products and coatings since their hydrophilic nature can cause them to separate or leak from substrates. Therefore, it’s critical to comprehend how graphene and its derivatives react in humid, corrosive microenvironments in biomaterials because the particles could damage tissues and organs if they enter the bloodstream (Liang et al., 2015; Volkov et al., 2017).

## 7 Conclusion

Materials made of graphene have various benefits for the dentistry industries. Many difficulties did exist, nonetheless, and they must be resolved. Overall, we should state that the application of graphene-based nanomaterials in dental domains merits careful study and has the potential to introduce a completely novel dental therapy conception henceforth.
